# Transposable Elements in the Revealing of Polymorphism-Based Differences in the Seeds of Flax Varieties Grown in Remediated Chernobyl Area

**DOI:** 10.3390/plants11192567

**Published:** 2022-09-29

**Authors:** Jana Žiarovská, Ivana Speváková, Lucia Klongová, Silvia Farkasová, Namik Rashydow

**Affiliations:** 1 Institute of Plant and Environmental Sciences, Faculty of Agrobiology and Food Resources, Slovak University of Agriculture in Nitra, Tr. Andreja Hlinku 2, 94976 Nitra, Slovakia; 2 Research Centre of AgroBioTech, Slovak University of Agriculture in Nitra, Tr. Andreja Hlinku 2, 94976 Nitra, Slovakia; 3 Institute of Cell Biology and Genetic Engineering, National Academy of Sciences of Ukraine, 148 Acad. Zabolotnogo Avenue, 03143 Kyiv, Ukraine

**Keywords:** CNPP, ionizing radiation, stress, flax, transposons, retrotransposons

## Abstract

The nuclear reactor accident in Chernobyl, Ukraine, resulted in effects both locally and farther away. Most of the contaminated areas were the agricultural fields and forests. Experimental fields were established near Chernobyl—radioactively contaminated fields localized 5 km from Chernobyl Nuclear Power Plant as well as the remediated soil that is localized directly in the Chernobyl town. Two flax varieties growing under chronic exposition to ionizing radiation were used for this study—the local Ukrainian variety Kyivskyi and a commercial variety Bethune. The screening of the length polymorphism generated by transposable elements insertions were performed. All known types of common flax transposon, retrotransposons and iPBS approach were used. In the iPBS multiplex analyze, for the Kyivskyi variety, a unique addition was found in the seeds from the radioactive contaminated field and for the Bethune variety, a total of five amplicon additions were obtained and one deletion. For the TRIM Cassandra fingerprints, two amplicon additions were generated in the seeds from radioactive contaminated fields for the Bethune variety. In summary, the obtained data represent the genetic diversity between control and irradiated subgroups of flax seeds from Chernobyl area and the presence of activated transposable elements due to the irradiation stress.

## 1. Introduction

The nuclear reactor accident in Chernobyl, Ukraine, on 26 April 1986, created acute, short-term and subsequently long-term effects both locally and farther away. Most of the contaminated areas were the agricultural fields and forests. The explosion released a total of 7.4 × 1016 Bq of radionuclides into the environment [1]. Since the accident, the radiological situation in the exclusion zone has stabilized and the high-dose-rate acute radiation has been replaced with persistent low-dose-rate chronic exposure [2,3].

Ionizing radiation was the first identified mutagen and it causes a wide range of intragenic and intergenic mutative changes. The range of mutation events caused by ionizing radiation vary from simple base substitutions to single- and double-strand breaks of DNA. An increase in mutation rates and changes in the structure and functioning of the genome is a part of the complex reactions of an organism when exposed to ionizing radiations and special studies were reported for long-living plants, such as *Pinus sylvestris* L. [4]. The studies of the adaptation mechanisms of herbs to adverse radioactive habitat conditions are limited, but the increase in mutation rates in radioecological studies as one of the reactions of organisms to irradiation is documented for different herbaceous plants [5,6,7,8]. For wheat plants, different alterations were reported for microsatellite fingerprints, including gains and losses of repeats with the estimation of mutation rate in the exposed group of plants at the level 6.63 × 10^−3^ [5]. The relative sable adaptation was described for *Stellaria graminea*, *Leonurus quinquelobatus*, *Silene latifolia* and *Bromus intermis* natural populations at the East-Ural Radioactive Trace [6]. The adaptation process of the herbaceous plants to the ionizing radiation is summarized as involving a complex genomic answer in the epigenetic regulation of gene expression and genome stabilization [7] and one of the radio protector mechanisms of irradiation stress in plants is connected with the activation of the antioxidant systems in plants [8]. One of the specific reactions to the stress caused by ionizing radiation, the induced activity of transposable elements, is reported in the literature [9,10]. Here, the transposon and retrotransposon response for the ionizing radiation was analyzed in the genome of the common flax. From the perspective of a global representation, flax (*Linum usitatissimum* L.) is the third largest natural fiber crop and one of the five major oil crops in the world and its genome was reported as plastic [11,12,13].

Transposable elements are characterized as a comprehensive network of a mobilome of organisms and are characterized by an ability to change position in the plant genome [14]. Two major classes of transposable elements are reported, class I and class II [15]. Class I represents retrotransposons with the action of transposition through reverse transcription of RNA, and class II represents DNA transposons in which transposition does not involve RNA but a cut-and-paste mechanism, and several transposase enzymes [16]. Retrotransposons are further divided into the LTR (long terminal repeat) and non-LTR retrotransposon [17]. LTR retrotransposons are classified into two major groups, Copia and Gypsy, consisting of many families [18]. Ty-1 copia elements can spread randomly throughout the genome and are more often associated with genes than Ty3-gypsy elements [19,20,21]. Activation of different transposable elements is linked mainly to the stress factors [22], and especially intragenic and environmental stress factors may lead to genome destabilization [23]. In the case of common flax, more than 20% of its genome is represented by transposable elements [19,24]. The most common forms of flax transposable element are LTR (long terminal repeated) retrotransposons. Different types of Ty1-copia retrotransposons are inserted in the flax genome and were identified on the basis of their LTR similarity and gene domain conservation and were reported to be active in the flax genome, and the polymorphism generated by them is often correlated among closely related flax cultivars [19].

DNA and transposable-based markers have been successfully applied in different types of analysis of genetic variability of flax [25,26,27,28,29] or for the purposes of flax identification [30]. Different types of transposable elements are described in the genome of common flax that made it possible to prove the effect of stress conditions on the flax genomes by generating the polymorphic fingerprints. Their sequences are fully accessible in the public databases. A total of twelve partial sequences of FL1-12 are known for flax, as well as Cassandra retrotransposon and dLUTE transposable element [31,32]. Defective *Linum usitatissimum* transposable element (dLUTE) is the only confirmed transposon of this specie that was characterized [33]. It was identified in two spontaneous mutant alleles of the L6 flax rust resistance gene and thanks to the missing ORF, it is supposed to be nonautonomous. dLUTE is similar to the Ac group of plant transposons based on its sequences, because of the presence of imperfect long terminal repeats and insertions by the mode of site duplications [33,34]. These characteristics were used for the development of the marker technique for the analysis of the length polymorphism of dLUTE insertions in the genome of common flax [35]. Besides dLUTE itself, dLUTE transposon similar sequences were reported as abundant in the flax genome [33]. Cassandra is a nonautonomous retrotransposon terminal repeat in miniature (TRIM), and similarly to other retrotransposons, is an active part of the genome of plants. This element was used for marker profiling in different plant species as well as for common flax [36]. It is organized in multiple extended tandem arrays within different plant species [37].

In this study, the changes of retrotransposon-based profiles of flax were analyzed through the insertional polymorphism of amplicons generated by the activation of transposable elements in two flax varieties, that were cultivated in the remediated and radio-contaminated area near the Chernobyl Nuclear Power Plant in the conditions of chronically ionizing radiation.

## 2. Results

Four different marker techniques were used that are based on the transposable elements generated via polymorphism to obtain specific patterns of their activity under the chronic radiation stress that is present in the area of Chernobyl—the inter prime binding site technique, inter retrotransposon polymorphism amplification technique, Cassandra-based length polymorphism technique, and dLUTE-based length polymorphism technique.

In the iPBS multiplex analysis, a total of 15 amplicons were generated for the Kyivskyi flax variety and 18 amplicons for Bethune variety. The length of the obtained amplicons varied from 177 bp up to the 1665 bp for the Kyivskyi and from 185 bp up to the 1635 bp for the Bethune variety.

**Figure 1:**
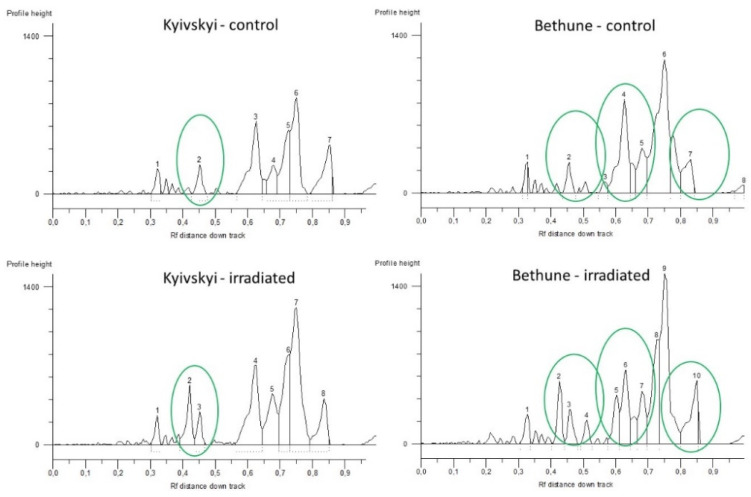
Comparison of obtained iPBS profiles of common flax varieties Kyivskyi and Bethune grown in the remediated and radio-contaminated field conditions of Chernobyl. x axis—Rf distance down track; y axis—profile height. 1–10—numbering of the generated loci.

In both of the analyzed varieties, different iPBS profiles were generated for the control seeds and for the seeds from plants grown in the radioactive contaminated fields (Figure 1). For the Kyivskyi variety, a unique addition of 1170 bp long amplicon was found in the seeds from the radio-contaminated field. For the Bethune variety, a total of five amplicon additions (185 bp, 380 bp, 620 bp, 855 bp and 1150 bp) were obtained, along with one deletion of the 700 bp long amplicon.

For the TRIM Cassandra fingerprints, a total of 17 amplicons were generated for the Kyivskyi flax variety and 19 amplicons for Bethune variety. The length of the obtained amplicons varied from 192 bp up to the 2282 bp for the Kyivskyi and from 212 bp up to the 2282 bp for the Bethune variety.

**Figure 2:**
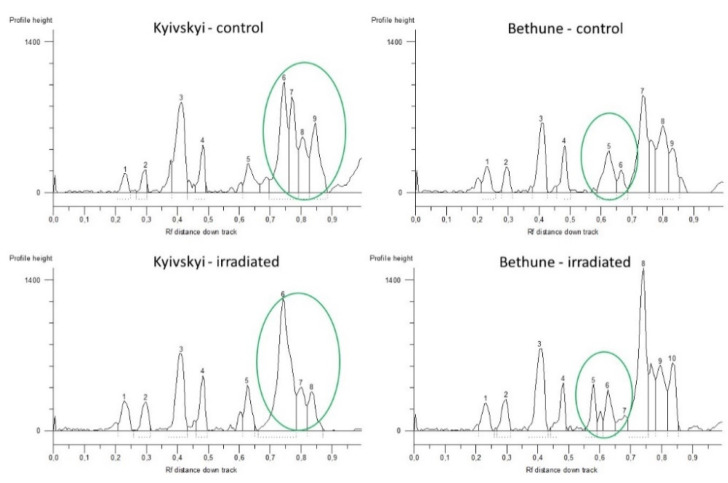
Comparison of obtained TRIM Cassandra profiles of common flax varieties Kyivskyi and Bethune grown in the remediated and radio-contaminated field conditions of Chernobyl. x axis—Rf distance down track; y axis—profile height. 1–10—numbering of the generated loci.

Different fingerprint profiles were generated for the control seeds and for the seeds from plants grown in the radioactive contaminated fields (Figure 2) by Cassandra-based marker. For the Kyivskyi variety, a different profile was amplified between the control and irradiated seeds in the small amplicon spectrum, where deletion of 300 bp can be seen. For the Bethune variety, a two amplicon additions −476 bp and 665 bp were generated in the seeds from radioactive contaminated fields.

**Figure 3:**
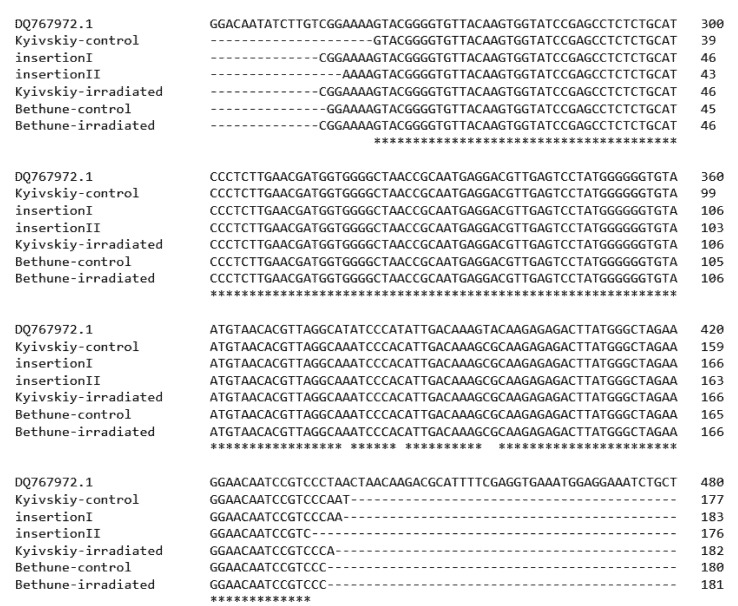
Sequence alignment by Clustal Omega of Cassandra amplicons generated in selected TRIM Cassandra fingerprints of common flax varieties Kyivskyi and Bethune grown in the remediated and radio-contaminated field conditions of Chernobyl.

Both of the new additions obtained in the Cassandra profile, as well as four randomly chosen amplicons (one for each analyzed accession) were checked for their sequence specificity by direct sequencing. The generated amplicons were resolved from agarose gels and checked. All of the obtained sequences were concordant with the Cassandra TRIM element sequence stored (DQ767972.1) in NCBI (Figure 3).

**Figure 4:**
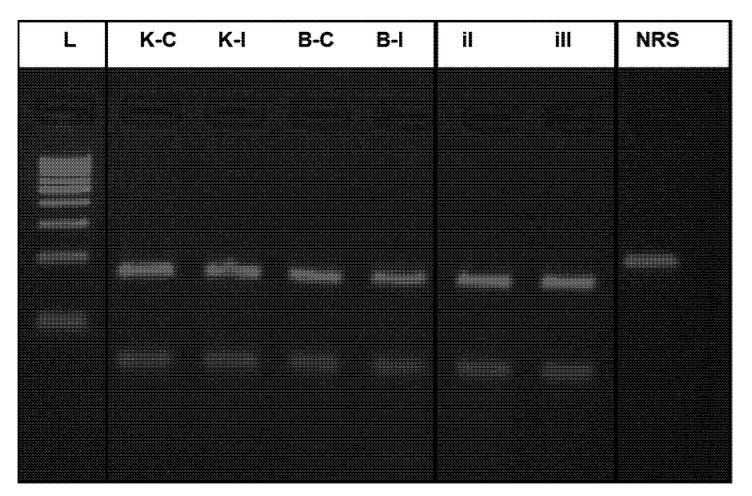
Restriction digestion of sequenced selected TRIM Cassandra fingerprints of common flax varieties Kyivskyi and Bethune grown in the remediated and radio-contaminated field conditions of Chernobyl. L—ladder (100 bp; Thermo Scientific); K-C: Kyivskyi control; K-I: Kyivskyi irradiated; B-C: Bethune control; B-I: Bethune irradiated; iI—inserted amplicon I; iII—inserted amplicon II; NRS—no restriction sample. A 1000 bp ladder was used (Fermentas).

The subsequent confirmation was performed by the RFLP analysis using the *RsaI* restriction enzyme (Figure 4). Two restriction products were obtained as was predicted in the in silico restriction module with length 177 + 39 bp for all of checked samples.

**Figure 5:**
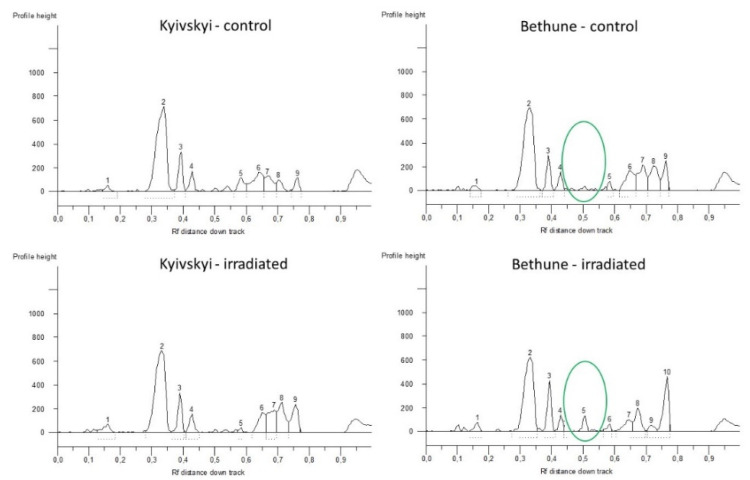
Comparison of obtained dLUTE profiles of common flax varieties Kyivskyi and Bethune grown in the remediated and radio-contaminated field conditions of Chernobyl. x axis—Rf distance down track; y axis—profile height. 1–10—numbering of the generated loci.

For the dLUTE transposon fingerprints, a total of 18 amplicons were generated for the Kyivskyi flax variety and 19 amplicons for the Bethune variety. The length of the obtained amplicons varied from 230 bp up to the 2090 bp for the Kyivskyi and from 220 bp up to the 2090 bp for the Bethune variety. The dLUTE profile that was obtained generated the most stable fingerprints among the length polymorphism techniques used, based on the transposable elements among the compared variants (Figure 5).

No specific changes were found for the Kyivskyi variety. For the Bethune variety, a unique amplicon addition of the length of 850 bp was generated in the seeds from the radioactive contaminated fields that was not amplified in the control variety.

**Figure 6:**
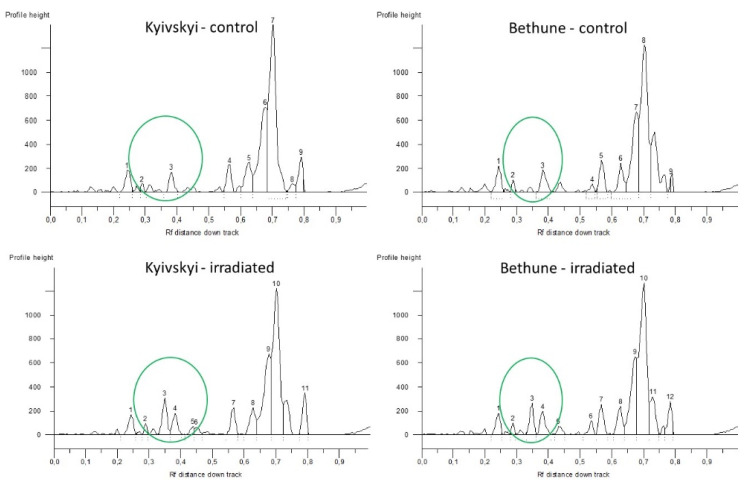
Comparison of obtained IRAP profiles of common flax varieties Kyivskyi and Bethune grown in the remediated and radio-contaminated field conditions of Chernobyl. x axis—Rf distance down track; y axis—profile height. 1–12—numbering of the generated loci.

In the IRAP multiplex analysis, a total of 20 amplicons were generated for the Kyivskyi flax variety and 21 amplicons for the Bethune variety. The length of the obtained amplicons varied from 192 bp up to the 1606 bp for the Kyivskyi and from 200 bp up to the 1606 bp for the Bethune variety. In both of the analyzed varieties, different IRAP profiles were generated for the control seeds and for the seeds from plants grown in the radioactive contaminated fields (Figure 6). Changes in amplicons between the control and irradiated seeds in the long amplicon spectrum were obtained. For the Kyivskyi variety, additions of 1150 bp, 867 bp and 837 bp long amplicon were found in the seeds from the radio-contaminated field. For the Bethune variety, a total of three amplicon additions (1150 bp, 883 bp and 276 bp) were obtained. A length difference was obtained for 622 bp/633 bp amplicon in the seeds from the control remediated field/contaminated field.

IRAP profiles revealed higher insertional polymorphism for the analyzed control and irradiated flax seeds when compared to the iPBS profiles, in spite of the fact, that both of the techniques anneal the retrotransposon sequences during the PCR. The main difference is in the specie-specific design of the IRAP primers to the LTR sites of retrotransposons that are active in the plant genomes, iPBS primers are designed to the evolutionary older PBS (primer binding site) sequences [38].

The iPBS and TRIM Cassandra-based fingerprints were similar in polymorphic information content calculation with the values of 0.38 and 0.37, and dLUTE with IRAP have similar values of 0.21 and 0.24, respectively. All of these values indicate that retrotransposon-based DNA markers amplify informative loci for flax genome when analyzed under the stress conditions. The polymorphic band percentage that was generated by these techniques reached the value of 100% for iPBS and TRIM Cassandra markers, 75% for dLUTE and 80% for IRAP. The values of discrimination power were: 5.8 for iPBS, 6 for TRIM Cassandra, 7 for dLUTE and 6.7 for IRAP, which indicate the comparability of used marker techniques for the analysis of whole genome polymorphism generated by transposable elements.

## 3. Discussion

Long-term mutagenic impact has become increasingly topical [39] because a new concept of radiation protection for humans and biota must be developed on the basis of a clear understanding of the nature of the formation of the biological effects of low-dose ionizing radiation [2]. Plant response to the radiation has been analyzed before by different techniques. Mutation rates of microsatellites in the Scots pine genome is reported as about 10^−4^ and a level of spontaneous mutations as revealed by AFLP as 10^−3^ per locus per generation [4]. Another studies of *Pinus sylvestris* growing in the Chernobyl area reported induced cytogenetic and genetic aberrations as a result of acute and chronic exposure of Scots pine trees to ionizing radiation [40]. Genomic DNA of the same specie was found to be hypermethylated in exposed trees and the degree of methylation seems to depend on the radiation dose absorbed by trees. Analysis of the mutation rate of wheat planted in Chernobyl area resulted in a six-fold increase over a single generation after the exposure to ionizing radiation [41,42]. Increased lethal mutations are reported for both, greenhouse and field populations of *Arabidopsis thaliana* in this environment [43]. Aberrant cells increased in a dose-dependent manner for wheat and rye in ionizing radiation [2]. Chronic irradiation at low doses reveals in the study [8] the dependence of changing the radio sensitivity of higher plants in the minimum on chronic β and γ irradiation, the number of chromosomes in the karyotype, and the advance γ irradiation with different dose fractioning, and for perennial plant populations, a significant increase in the frequency of mutations was reported.

Transposable elements are a significant contributor to the genome variability and plasticity in plant species, mainly as plants cannot hide from environmental stress, and are forced to build a complex network of adaptation processes [44]. Different authors have referred to the retrotransposons stress activation and its role in plant defense and adaptation mechanisms [45,46]. Retrotransposon induction by stress can lead to insertional or deleterious mutations that cause the genetic instability or alterations in their insertions that accomplish a protective function in plant genomes [47].

The Chernobyl area is contaminated with long-living radioisotopes including ^90^Sr and ^137^Cs [48], nevertheless, activity toward the tendency of the remediation of the contaminated areas for the agriculture purposes started here [49]. The local ecosystem has been able to adapt to a constantly high level of radiation and for this reason, to evaluate plant adaptation and response, seeds of a local flax variety Kyivskyi and a commercial variety Bethune were sown in radio-contaminated and control fields of the Chernobyl region [50]. Flax was selected because it is a crop of economic and historical importance, despite the relative paucity of molecular resources [49,50]. Moreover, the flax genome is reported to have very different plasticity in the reactions to environment stimuli in the literature [51] and the background of this plasticity and genomic changes may originate in different and not-fully-understood mechanisms of response to stress, such as the activation of transposable elements [52].

In this study, flax (*Linum usitatissimum* L.) cultivated in the Chernobyl area was analyzed. The DNA isolated from the samples from control and radioactive zone was applied for the screening of transposons and retrotransposons activity analyzed by DNA-based markers. A bulk DNA was used for individual accessions. This strategy can lead to different band density of some markers obtained from bulked accessions and individual accessions, but when optimizing the repeatability of the used technique, is fully reproducible as was reported for RAPD before [52,53], and is one of the most sensitive methods in the meaning of reproducibility. Of all of the techniques used here, only repeatable and unambiguous amplicons were scored.

Retrotransposon-based techniques were utilized here to describe the flax variety variability under the stress. Individual iPBS markers (2074, 2080, 2228, 2230, 2232, 2249 and 2251) were used previously to analyze the changes in both of the analyzed flax varieties under the ionizing radiation [38]. Based on the results in this study, where specific IRAP markers were more sufficient to reveal retrotransposon activation in response to radionuclide contamination, only the selected iPBS markers were used here in a multiplex PCR to compare all of transposon-based techniques. The molecular response of flax to the chronic low-dose radiation was shown by generated insertional polymorphism. All of the used iPBS primers generate the variable profiles and some of them yielded solely monomorphic profiles. Those with monomorphic profiles were not used for further analysis.

Transposable elements were used for the developing of marker techniques for the flax analysis, too. Previously, an in silico approach aimed at developing dLUTE primers for length polymorphism analysis, and also at generating polymorphic profiles for 24 different flax varieties [35].

Retrotransposon-based techniques provide a powerful tool for the analysis of different aspects of natural genomic variability of higher plants and were used for many plant species [54]. When comparing the individual techniques used in this study, all of them generated insertional polymorphism in the flax seeds harvested from the radio-contaminated fields. Amplification efficiency in both of the varieties was comparable for all of the used techniques. Control variants of both analyzed flax varieties had the same fingerprints profiles except of IRAP. The dLUTE fingerprints were the most stable in amplification and the only fragment addition was obtained in the Bethune variety. In this flax variety, more changes were generated when compared to the Kyivskyi. Bethune referred to as a stress unresponsive variety in the literature, which does not exhibit any of the phenotypic plasticity that accompanies the rapid genome change observed in Stormont Cirrus, using the insertion of LIS-1 as the criteria for responsiveness [51,55,56]. What can be hypothesized is an environment-specific ability of local varieties to be more stable in the transposon/retrotransposon activity under the abiotic stress, but this must be investigated further. Retrotransposon markers show higher polymorphism in the native species, but in the case of flax the full-length retrotransposons corresponding to the IRAP primers were not identified and they appear to be both conserved and active in multiple Linum species, except the individual Cassandra-type TRIM retroelement, that show a higher level of polymorphism [57].

## 4. Materials and Methods

### 4.1. Plant Material and Experimental Field Conditions

Starting 2007, different experimental fields were established near Chernobyl aimed at identifying plant response to chronic radiation. The radioactively contaminated field is localized approximately 5 km from CNPP with the characteristics: ^137^Cs 20650 ± 1050 Bq.kg^−1^ and ^90^Sr 5180 ± 550 Bq.kg^−1^. The control field with the remediated soil is localized directly in the Chernobyl town with the characteristics: ^137^Cs 1414 ± 71Bq.kg^−1^; and ^90^Sr 550 ± 55 Bq.kg^−1^. Harvested flax seeds’ radioactivity uptake from radioactive contaminated field has been determined as 780 ± 39 Bq.kg^−1^ of ^137^Cs and 3550 ± 360 Bq.kg-1 of ^90^Sr. For seeds from control field, radioactivity has been determined as 8 ± 5Bq.kg^−1^ of ^137^Cs and 90 ± 16 Bq.kg^−1^ of ^90^Sr.

In this study, two flax varieties were used for analysis. Both of them were grown in control as well as in contaminated fields. Mature seeds for this study were harvested at the end of the 2012 growing season. The local Ukrainian variety Kyivskyi used here was the 6th generation cultivated in experimental fields and a commercial variety Bethune was added to the comparison as the 1st generation. Both of them are oilseed genotypes. No specific treated seeds were sown in the remediated and experimental fields in May. All of the biological material were collected in a triplicate and was chronically exposed to ionizing radiation during its growing.

### 4.2. DNA Extraction

DNA extraction from the mature flax seeds was performed from 0.5 g of dry flax seeds that was homogenized using TissueLyzer II (Qiagen). DNA was isolated by Rogers and Bendich protocol [56] following the original instructions and the DNA pellet was rehydrated in 500 mL of 0.1 × TE. DNA was isolated as bulk one and analysis were performed in technical triplicates after the dilution of DNA of all accessions to uniform 100 ng/µL.

### 4.3. Transposable Elements Profiling

The screening of the length polymorphism generated by transposable elements insertions were performed. All known types of common flax transposon, retrotransposons and iPBS approach were used.

IRAP method was performed by primers as described in [32] where the following IRAP primers were chosen for the analysis: 1826, 1833, 1838, 1854 and 1868. Multiplex conditions of the reaction were optimized for the annealing temperature and the final 58 °C was used in the analysis using a FIREPol DNA polymerase (Solis BioDyne, Tartu, Estonia)—2 U and 1200 nmol of primer mix where all of the primers were in the equally content. PCR amplification was performed in 15 µL using a SureCycler 8800 Thermal Cycler (Agilent Technologies, Santa Clara, CA, USA). The cycling program was of following parameters: 5 min initial denaturation at 95 °C; 35 cycles of 95 °C denaturation for 45 s; 58 °C primer annealing for 35 s; 72 °C elongation for 4 min and the final elongation for 10 min at 72 °C. Amplicons were separated by 1.8% agarose electrophoresis SERVA Electrophoresis GmbH, Germany) in a TBE buffer stained by GelRed™ (Biotium, Fremont, CA, USA) for UV visualization.

The iPBS method was performed by using the primers as described in [32]. Five different iPBS primers were used in the multiplex analysis: 2230, 2249, 2251 and 2080. These primers were selected based on our previous results, and all of them were used individually and tested for their discrimination ability for the analyzed varieties [38]. Primer annealing temperature was optimized in the gradients and the final 50 °C was used in the analysis. FIREPol DNA polymerase (Solis BioDyne)—2 U and 1500 nmol of primer (all of the primers were in the equally content) was used in the PCR and the amplification was performed in 15 µL using a SureCycler 8800 Thermal Cycler (Agilent). The cycling program was of the following parameters: 5 min initial denaturation at 95 °C;—35 cycles of 95 °C denaturation for 45 s; 50 °C primer annealing for 30 s; 72 °C elongation for 3 min and the final elongation for 10 min at 72 °C. Amplicons were separated by 1.8% agarose electrophoresis (Serva) in a TBE buffer stained by GelRed™ (Biotium) for UV visualization.

Polymorphism generated by dLUTE flax transposon was performed by primers according the [35]. DreamTaq DNA polymerase (Thermo Fisher Scientific, Waltham, MA, USA) was used in the PCR and the amplification was performed in 10 µL using a SureCycler 8800 Thermal Cycler (Agilent). The cycling program was of following parameters: 3 min initial denaturation at 95 °C; 40 cycles of 95 °C denaturation for 45 s; 54 °C primer annealing for 40 s; 72 °C elongation for 2 min and the final elongation for 10 min at 72 °C. Amplicons were separated by 15% PAGE electrophoresis in a TBE buffer stained by GelRed™ (Biotium) for UV visualization.

Polymorphism generated by Cassandra TRIM retrotransposon was evaluated by primers as reported for flax previously [58]. DreamTaq DNA polymerase (Thermo Fisher Scientific) was used in the PCR and the amplification was performed in 10 µL using a SureCycler 8800 Thermal Cycler (Agilent). The cycling program was of following parameters: 2 min initial denaturation at 94 °C; 35 cycles of 94 °C denaturation for 60 s; 54 °C primer annealing for 60 s; 72 °C elongation for 2 min and the final elongation for 10 min at 72 °C. Amplicons were separated by 1.5% agarose electrophoresis (Serva) in a TBE buffer. Gels were stained by GelRed™ (Biotium) and digitally photographed.

**Figure 7:**
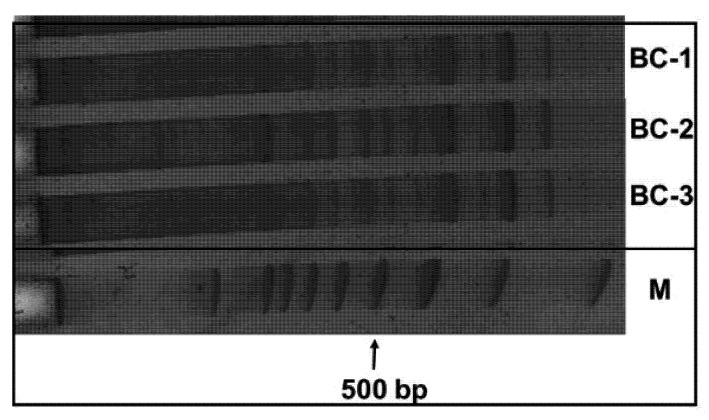
Cassandra profile of Bethune control variant in triplicate. A 1000 bp ladder was used (Fermentas).

DNA markers analysis were performed in triplicates (Figure 7).

### 4.4. Cassandra TRIM Element Specifity Checking

A 216 bp product of Cassandra internal domain was amplified using sequencing PCR in the C1000 thermocycler (Bio-Rad, Hercules, CA, USA). The 25 µL reaction mixture contained 1.5 mM MgCl_2_, 200 µM of each dNTP, 0.4 µM of each primer, 1 × MyTaq PCR buffer, 1 U MyTaq DNA HS polymerase (Bioline, London, UK) and 50 ng of genomic DNA template. Thermal cycling conditions included: an initial denaturation step at 95 °C for 3 min followed by 30 cycles of 95 °C for 5 s, 62 °C for 20 s, 72 °C for 30 s and a final extension for 5 min at 72 °C.

The PCR products were amplified with the PCR primers designed in Primer Blast to the sequence of Cassandra TRIM of flax in the NCBI (DQ767972) with the following sequences: forward 5′GGGATTAGTTATGCCCAAACCGGAC3′ and reverse 5′GGGACGGATTGTTCCTTCTAGCCC3′). The reaction mixture in a total volume of 20 µL contained 1×Phusion^®^High-Fidelity PCR Master Mix (Finnzymes, Espoo, Finland), 0.4 µM of each primer and 10 ng of genomic DNA.

After amplification the PCR products were purified using 1.5 IU FastAPTM Thermosensitive Alkaline Phosphatase (Fermentas GmbH, Waltham, MA, USA) and 10 IU Exonuclease I (Fermentas GmbH) according to the manufacturer´s protocol. The purified PCR products were sequencing in both directions by the GenomeLabTM DTCS (Beckman Coulter, Brea, CA, USA) sequencing kit. The sequencing analysis was performed on an eight-capillary sequencer GenomeLab GeXPTM Genetic Analysis System (Beckman Coulter, Brea, CA, USA). The generated sequencing data was not stored in the public sequence databases, as they were used for specificity checking of known sequence.

The confirmation of restriction site for an endonuclease of the PCR products was performed 30 min with 1 µL of the FastDigest *RsaI* restriction enzyme (Fermentas GmbH) at 37 °C. The identification of restriction fragments was performed in 2% agarose gel (SERVA Electrophoresis GmbH, Heidelberg, Germany) with an intercalation reagent GelRedTM (Biotium, Fremont, CA, USA) in 1×TBE buffer at 130 V for 30 min.

### 4.5. Data Analysis

Clear and unambiguous amplicons generated by used techniques were recorded using the SynGene GeneTools v 4.01.04 software for gel image analysis. A 100 bp ladder (Thermo Scientific) was used for amplicons length determination. The presence (1) or absence (0) of the obtained amplicons between the controls and irradiated conditions were compared for both of the used flax varieties and a qualitative analysis was performed for the amplicons. PIC (polymorphic information content) was calculated according to De Riek et al. [59] (2001). DP (discrimination power) was calculated according the Tessier et al. [60] (1999).

## 5. Conclusions

The mechanism of retrotransposons’ action toward plant plasticity in radio-contaminated environment is very limited in our knowledge. This study reports the DNA-based generated polymorphism of insertional changes of flax transposon/retrotransposon, with evidence that the alterations are present on the genetic level for a long period after the initial release of radioactivity into the environment. I summary, the data represent the genetic diversity between control and irradiated subgroups of flax seeds from Chernobyl area and the presence of activated transposable elements due to the irradiation stress.
